# Phosphoprotein Gene Contributes to the Enhanced Apoptosis Induced by Wild-Type Rabies Virus GD-SH-01 *In Vitro*

**DOI:** 10.3389/fmicb.2017.01697

**Published:** 2017-09-05

**Authors:** Qin Tian, Yifei Wang, Qiong Zhang, Jun Luo, He Jiang, Boyue Zhang, Mingzhu Mei, Fan Wu, Yuting Wu, Jiaojiao Peng, Teng Long, Yongwen Luo, Xiaofeng Guo

**Affiliations:** ^1^College of Veterinary Medicine, South China Agricultural University Guangzhou, China; ^2^Key Laboratory of Zoonosis Prevention and Control of Guangdong Province Guangzhou, China

**Keywords:** rabies virus, phosphoprotein, apoptosis, GD-SH-01, HEP-Flury, chimeric viruses, intrinsic apoptotic pathway

## Abstract

Previous research demonstrated that the matrix protein (M) and glycoprotein (G) of attenuated rabies virus (RABV) strains are involved in the induction of host cell apoptosis. In this work, we show that wild-type (wt) RABV GD-SH-01 induces significantly greater apoptosis than the attenuated strain HEP-Flury. In order to identify the gene(s) accounting for this phenotype, five recombinant RABVs (rRABVs) were constructed by replacing each single gene of HEP-Flury with the corresponding gene of GD-SH-01. By using these rRABVs, we found that not only M and G, but also the phosphoprotein (P) plays an important role in inducing apoptosis. In order to figure out the different role of P gene in inducing apoptosis from the highly divergent background, another rRABV rGDSH-P, which carries the P gene of HEP-Flury in the background of the GD-SH-01 was generated. It was found that infection of NA cells with GD-SH-01 or the recombinant strain rHEP-shP, which carries P gene of GD-SH-01, induced significantly greater apoptosis than HEP-Flury or rGDSH-P in a caspase-dependent pathway that ultimately leads to the activation of the intrinsic apoptotic pathway, which is well characterized with the downregulation of bcl-2, the decrease of mitochondrial membrane potential, the release of mitochondrial cytochrome c, the activation of caspase-9 and caspase-3, and finally the cleavage of poly (ADP-ribose) polymerase. Our results imply that wt P from GD-SH-01 mediates this effect may partly by facilitating viral RNA synthesis but not by viral replication. In sum, we demonstrate a wt RABV strain GD-SH-01 to induce stronger apoptosis than an attenuated RABV HEP-Flury and propose that wt P from GD-SH-01 is involved in this process.

## Introduction

The process of apoptosis is often referred to as programmed cell death and has first been described by John Kerr in 1972 (Kerr et al., 1972). It is an evolutionarily conserved form of cell suicide that requires a specialized machinery in which the main components are specific proteases, namely caspases (Thornberry and Lazebnik, 1998). To date, research indicates that there are two main apoptotic pathways that both involve the activation of caspases: the intrinsic or mitochondrial pathway and the extrinsic or death receptor pathway. In detail, the former has its origin in the mitochondrion and is triggered by a variety of intracellular signals that cause mitochondrial proteins like cytochrome c to be released into the cytoplasm. Cytochrome c colocalizes with caspase-9 and Apaf-1 and forms the so-called apoptosome. Caspase-9 is activated within the apoptosome and subsequently mediates the activation of executioner caspase-3. This caspase effectuates the cleavage and inactivation of poly (ADP-ribose) polymerase (PARP) and thereby induces apoptosis (Sun et al., 1999; Jiang and Wang, 2004; Sarmento et al., 2006). In contrast, the extrinsic apoptotic pathway is initiated by the activation of membrane-bound death receptors, such as Fas receptor and tumor necrosis factor (TNF-α) receptor. These receptors mediate the activation of caspase-8, which, in turn, activate caspase-3, that cleaves its substrates and induces apoptosis as described above (Miller, 1997; Ashkenazi and Dixit, 1998). Host cell apoptosis can be beneficial or detrimental for the virus, as it constitutes a means of release and dissemination of progeny viruses, but also a defense strategy pursued by the host to counteract the viral infection (Mori et al., 2004). Apoptosis has been shown to occur upon infection with several viral pathogens, e.g., influenza A virus, variant H3N2 (Takizawa et al., 1993), herpes simplex virus 1 (Galvan and Roizman, 1998), human immunodeficiency virus (Cummins and Badley, 2010), and rabies virus (RABV) (Baloul and Lafon, 2003).

Rabies virus is the etiologic agent of rabies, which still causes tens of thousands of deaths every year (World Health Organization, 2010). As a member of the genus *Lyssavirus* in the family of Rhabdoviridae, RABV is a neurotropic virus that causes fatal encephalitis in warm-blooded animals and humans (Jackson, 2003). This virus has a non-segmented, single-stranded, negative-sense RNA genome of approximately 12 kb, which comprises five genes that encode the nucleoprotein (N), the phosphoprotein (P), the matrix protein (M), the glycoprotein (G), and the RNA-dependent RNA polymerase, which is also referred to as large protein (L), in the order of 3′-N-P-M-G-L-5′ (Tordo et al., 1986). In order to gain a better understanding of the molecular mechanisms underlying RABV-induced apoptosis, extensive research has been conducted on the role of apoptosis in RABV infections both *in vitro* and *in vivo*. Some studies have been focused on the pathogenic traits displayed by adapted RABV strains, such as Challenge Virus Standard-11 (CVS-11) and ERA, while other groups have worked with wild-type (wt) RABV strains like SHBRV-18 (Thoulouze et al., 1997; Baloul and Lafon, 2003; Prehaud et al., 2003; Sarmento et al., 2006). Previous research has also demonstrated laboratory-attenuated RABV strains to induce apoptosis, a process that has mainly been attributed to proteins G and M (Yan et al., 2001; Kassis et al., 2004; Zan et al., 2016), in contrast, wt RABV did not display this pro-apoptotic phenotype. However, according to our published work, wt RABV GD-SH-01 does induce greater apoptosis in mouse neuroblastoma (NA) cells than the attenuated RABV HEP-Flury (Peng et al., 2016). These results are not entirely consistent with findings reported in the past (Thoulouze et al., 1997; Yan et al., 2001), although some reports do support our results (Ubol and Kasisith, 2000; Ubol et al., 2005; Yin et al., 2014). This apparent contradiction may be due to strain-specific traits (Peng et al., 2016), although evidence to this end is still scarce (Prabhavathy and Palanivel, 2015).

In order to identify the mechanisms underlying phenotypic differences between attenuated HEP-Flury and wt GD-SH-01 and to clarify which genes of wt GD-SH-01 might play a role in the enhanced apoptosis induction, a series of recombinant RABV (rRABV) strains was constructed. For each rRABV, single genes of HEP-Flury have been replaced with the corresponding genes of GD-SH-01 (Tian et al., 2017), in addition, another rRABV rGDSH-P was generated by replacing the P gene of GD-SH-01 with that of HEP-Flury. The present study initially aimed at confirming wt RABV GD-SH-01 strain’s stronger ability to induce apoptosis in NA cells when compared with HEP-Flury, but also at demonstrating that in addition to M and G, as has been implied before (Li et al., 1998; Kammouni et al., 2015), P of GD-SH-01 is also required for the pro-apoptotic phenotype. Further experiments revealed that P gene of GD-SH-01 was involved in the activation of the intrinsic apoptotic pathway and that infections with GD-SH-01 or rHEP-shP lead to the downregulation of bcl-2, the decrease of mitochondrial membrane potential (MMP), the release of mitochondrial cytochrome c, the activation of caspase-9 and caspase-3, and finally the cleavage of PARP. However, P of neither GD-SH-01 nor HEP-Flury was able to induce detectable apoptosis. To our knowledge, this is the first study providing evidence that P gene of RABV plays a role in host cell apoptosis after viral infection. The results obtained in this study will extend our knowledge of RABV-induced apoptosis, which will help to understand the pathogenic mechanism of wt RABV since apoptosis are always closely correlated to viral pathogenicity and immunogenicity (Theerasurakarn and Ubol, 1998; Morimoto et al., 1999; Pulmanausahakul et al., 2001), and provide new perspectives to the pathogenic mechanism of virulent RABV.

## Materials and Methods

### Cells and Viruses

**FIGURE 1:**
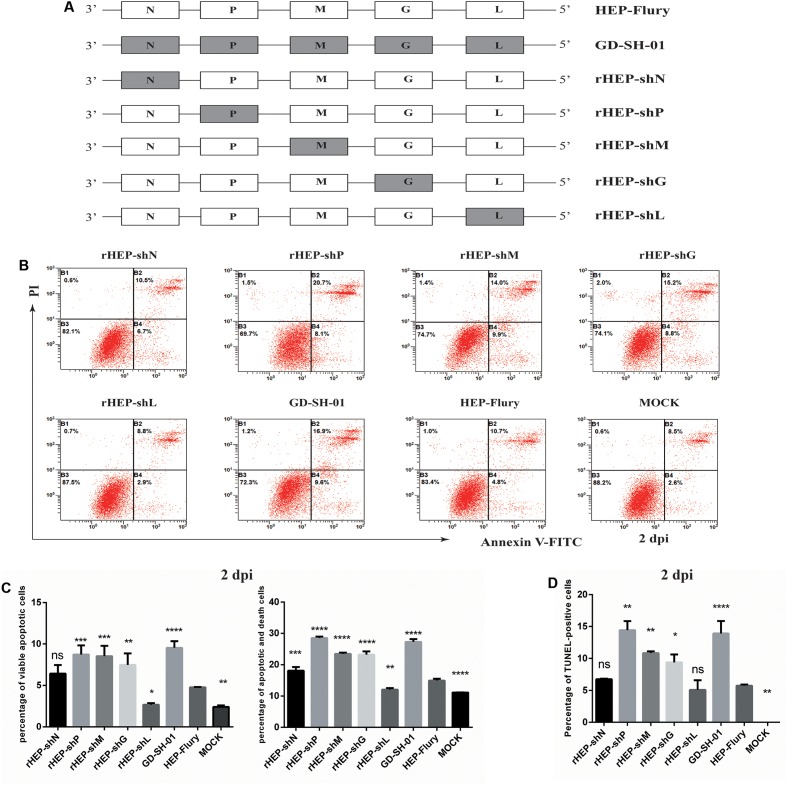
Wild-type GD-SH-01 induces significantly greater apoptosis than attenuated HEP-Flury. **(A)** Schematic representations of the gene order of GD-SH-01, HEP-Flury, and rRABVs. **(B)** NA cells were infected with GD-SH-01, HEP-Flury, or chimeric viruses. Cells in early stage apoptosis are Annexin V^+^/PI^-^, while dead cells correspond to those marked Annexin V^+^. **(C)** Chart plot of host cell apoptosis at 2 dpi. **(D)** Percentages of TUNEL-positive cells at 2 dpi. Data are represented as means ± SD of three independent experiments. One-way ANOVA followed by Bonferroni’s multiple comparison test. Comparing with HEP-Flury, ^∗^*p* < 0.05, ^∗∗^*p* < 0.01, ^∗∗∗^*p* < 0.001, and ns, not significant.

**FIGURE 2:**
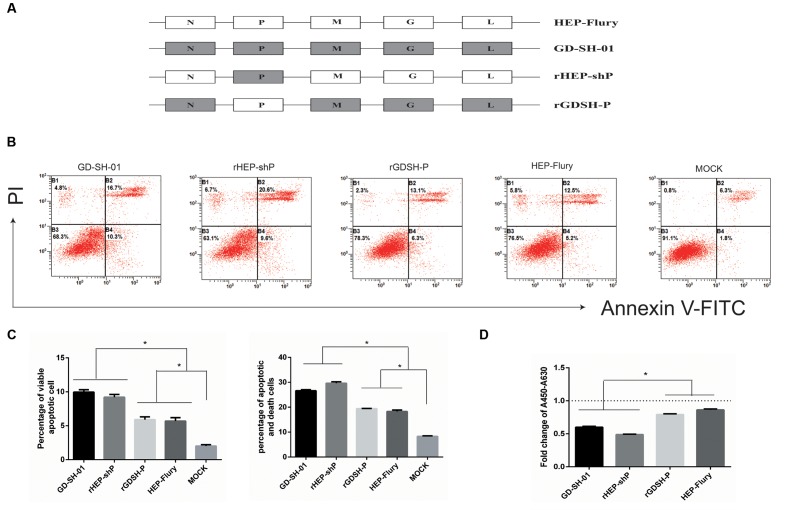
P gene of GD-SH-01 contributes to the enhanced apoptosis induced by wt GD-SH-01. **(A)** Schematic representations of the gene order of GD-SH-01, HEP-Flury, rHEP-shP, and rGDSH-P. **(B)** NA cells were infected with GD-SH-01, HEP-Flury, rHEP-shP, or rGDSH-P. Cells in early stage apoptosis are Annexin V^+^/PI^-^, while dead cells correspond to those marked Annexin V^+^. **(C)** Chart plot of host cell apoptosis at 2 dpi. **(D)** NA cells were infected with GD-SH-01, HEP-Flury, rHEP-shP, or rGDSH-P, and the cell viability was determined. Data are represented as means ± SD of three independent experiments. One-way ANOVA followed by Bonferroni’s multiple comparison test. ^∗^*p* < 0.05.

Neuroblastoma cells of A/J mouse origin were maintained in RPMI 1640 medium (Gibco, China) supplemented with 10% FBS, at 37°C and 5% CO_2_. The HEP-Flury strain was preserved in our laboratory after it had been rescued (Yang et al., 2014). The wt strain GD-SH-01 was isolated from a rabid pig in our laboratory (Luo et al., 2012). Chimeric RABVs carrying single genes of GD-SH-01 in the background of the HEP-Flury genome (rHEP-shN, rHEP-shP, rHEP-shM, rHEP-shG, and rHEP-shL, which is outlined in **Figure 1A**) were generated by a reverse-genetics approach (Tian et al., 2017). In addition, another rRABV rGDSH-P carrying the P gene of HEP-Flury in the background of the GD-SH-01 (**Figure 2A**) was also generated via the reverse-genetics system based on GD-SH-01, and the primers used to construct rGDSH-P is listed in **Table 1**. All virus stocks were prepared in NA cells and stored at -80°C. Viruses were titrated in NA cells using fluorescein isothiocyanate-conjugated antibodies directed against N of RABV (FujiRebio Diagnostic Inc., PA, United States), as described previously (Mei et al., 2017).

**Table 1:** Sequence of primers used to assembly full-length genome cDNA of rGDSH-P^a^.

Target	Primers	Primer sequence (5′–3′)	Length (bp)
Linearized vector	VP1	CAGGCAACACCACTGATAAGATGAACTTCCTACGCAAGATAG	1073
	VP2	AGTTTTTTTCATGTGAGATATACACAATTTGCAGGTTCTTTTCAATCTC	
Inserted fragment	FP1	TATCTCACATGAAAAAAACTAACACTCCTCCTTTCGAACCATCCCAAGTATG	15,717
	FP2	CTTATCAGTGGTGTTGCCTGTTTTTTTCATATCGACTCCATGACATCTCAG	


*^a^20 bp homologous sequences are underlined.*

### Antibodies and Reagents

For Western blots, primary antibodies against bax (AF0120) and caspase-3 (AF6311) were purchased from Affinity Biosciences Inc. (OH, United States). The primary antibody against bcl-2 (2876) was obtained from Cell Signaling Technology, Inc. (MA, United States). The primary antibody against caspase-9 (ab184786) was bought from Abcam (Cambridge, MA, United States), while the anti-cytochrome c antibody (bs-0013R) was purchased from Beijing Biosynthesis Biotechnology Co., Ltd. (Beijing, China). The primary antibody against PARP (AP102), COX IV (AC610), and β-actin (AA128) was obtained from Beyotime (Beijing, China). All the above primary antibodies except β-actin are rabbit polyclonal antibodies while the latter is a mouse monoclonal antibody. Horseradish peroxidase-labeled AffiniPure goat anti-rabbit and anti-mouse secondary antibodies were purchased from Vazyme Biotech Co., Ltd. (Nanjing, China). Recombinant murine TNF-α (C600052) was purchased from Sangon Biotech Co., Ltd. (Shanghai, China).

### Virus Infection and One-Step Growth Assays

Neuroblastoma cells were grown to approximately 80% confluence and were then infected with the respective virus at a multiplicity of infection (MOI) of 2. For virus one-step growth assays, samples of cell culture supernatants were harvested daily during 5 days following the infection, and virus titers were determined by means of direct immunofluorescence assays (dFAs) as described before (Wang et al., 2014).

### Cell Viability Assay

To evaluate the viability of NA cells infected with GD-SH-01, HEP-Flury, or any rRABV strain, the WST-1 Cell Proliferation and Cytotoxicity Assay Kit (Beyotime, China) was employed according to the manufacturer’s instructions. Mock-infected NA cells were used as negative controls. In this setting, the optical density (OD) of prepared cell suspensions served as an indicator of cell counts and thus allowed for the deduction of cell proliferation and viability. The OD was determined at a wavelength of 450 nm, with measurements at 690 nm serving as blanks. Experiments were performed in a Model 680 Microplate Reader (Bio-Rad, CA, United States).

### Plasmid Construction and Cell Transfection

For the construction of a plasmid capable of expressing P of HEP-Flury, we utilized plasmid mRFP-shP as the backbone, which carries RFP gene to facilitate monitoring the efficacy of the transfection and expression levels of P under a fluorescence microscope (AMG, Mill Creek, WA, United States) (Peng et al., 2016). We replaced the P gene carried by mRFP-shP with that of HEP-Flury using the seamless cloning technology (Vazyme, Nanjing, China) and the primers listed in **Table 2**. Another plasmid carries the mRFP-shP backbone without shP was also constructed and served as the mock control. Cell transfection was conducted using the SuperFect Transfection Reagent (Qiagen, Germany) and cultured at 37°C for 24 h as previously described (Peng et al., 2016).

**Table 2:** Oligonucleotides used for construction of plasmid mRFP-P^a^.

Primers	Sequence (5′–3′)	Use
mRFP-P1	GAATTCTGCAGTCGACGGTACCGCG	Amplifying the linearized vector mRFP
mRFP-P2	CATGGTGGCGACCGGTCCACC	
HEP-P1	GTGGACCGGTCGCCACCATGAGCAAGATCTTTGTTAATCCGAGTGC	Amplifying the inserted HEP-P gene
HEP-P2	TACCGTCGACTGCAGAATTCTTAGCATGATGTGTAGCGATCCAAGTC	


*^a^20 bp homologous sequences are underlined.*

### Flow Cytometry

To evaluate the ability of distinct RABVs or viral proteins to induce apoptosis *in vitro*, NA cells were infected with RABVs (GD-SH-01, HEP-Flury, or individual rRABVs) or transfected with different plasmids expressing single viral proteins. At 1 and 2 days post-infection (dpi), cells were harvested and stained with Annexin-V and propidium iodide (PI) using the Annexin V-FITC/PI Kit (BestBio, China). Here, the ratio of apoptotic cells corresponded to the share of Annexin V^+^/PI^-^ cells and the ratio of death cells corresponded to the share of Annexin V^+^ cells. Flow cytometry (FCM) was performed using a Beckman FC500 flow cytometer (Beckman Coulter, MA, United States). Data were analyzed with the corresponding CXP analysis software (Beckman Coulter).

### TUNEL Assay

Terminal deoxynucleotidyl transferase-mediated nick end-labeling (TUNEL) assay was also performed to detect the apoptosis of NA cells infected with RABVs with One Step TUNEL Apoptosis Assay Kit (Beyotime, China). Briefly, cells seeded in 24-well plates were infected with each RABV or media alone, at 2 dpi, cells were washed once with phosphate-buffered saline (PBS), and fixed in 4% paraformaldehyde for 30 min at room temperature. After rinsing twice with PBS, cells were permeabilized using 0.3% Triton X-100 in PBS for 5 min at room temperature. Cells were then incubated with the TUNEL reaction mixture for 1 h at 37°C in a humidified atmosphere in the dark. Cells were then rinsed three times and examined with a fluorescence microscope (AMG, United States). Cells displaying green fluorescence were recorded as TUNEL-positive cells. The apoptotic index was calculated by analyzing the average percentages of TUNEL-positive cells in three random fields from different wells using a ×400 magnification.

### JC-1 Staining to Measure Mitochondrial Membrane Potential

To detect a change in MMP (or ΔΨm), NA cells were stained with 5,50,6,60-tetrachloro-1,10,3,30-tetraethyl-imida carbocyanine iodide (JC-1; Beyotime, China). Whether JC-1 forms monomers or polymers depends on the MMP. 5,50,6,60-Tetrachloro-1,10,3,30-tetraethyl-imidacarbocyanine iodide accumulates in the mitochondrial matrix to form red fluorescent J-aggregates in normal cells, in which the MMP is high. When cells undergo early apoptosis, the MMP is low, which prevents JC-1 accumulation in the mitochondria and thus, the dye is dispersed throughout the entire cell leading to a shift from red (JC-1 aggregates) to green fluorescence (JC-1 monomers) (Wang et al., 2010; Peng et al., 2016). Briefly, cells were cultured in 6-well plates. After infected at indicated time points, cells were stained with JC-1 (2 mM final concentration) and incubated in the incubator (37°C, 5% CO_2_) for 20 min. Following incubation, cells were washed once with PBS and then analyzed by FCM. In each sample, 30,000 events were measured after excitation at 514 and 585 nm were used for further analysis. Results are expressed as the percentage of NA cells with depolarized mitochondria (Green Fluorescence ^bright^, Red Fluorescence ^dim^).

### Caspase-3, -8, and -9 Activities Assay

Caspase-3, -8, and -9 activities were quantified using the Caspase 3 Activity Assay Kit (C1116), Caspase 8 Activity Assay Kit (C1152), and Caspase 9 Activity Assay Kit (C1158, all from Beyotime), according to the manufacturer’s protocols. Briefly, at 2 dpi, cells cultured in 6-well plates were collected and centrifuged at 600 × *g* at 4°C for 5 min, and then resuspended in ice-cold cell lysis buffer on ice for 15 min. Cell lysates were again centrifuged, at maximum speed and 4°C for 10 min, before the supernatant was collected. After the reaction buffer and corresponding caspase substrate were added, the mixture was incubated in a water bath at 37°C for 60 min. The substrates of caspase-3, -8, and -9 were Ac-DEVD-pNA, Ac-IETD-pNA, and Ac-LEHD-pNA, respectively. Absorption of the preparation at 405 nm directly correlated with the concentration of pNA and thus allowed us to calculate the activity of the respective enzyme. Activities are expressed as fold change over the activity detected in mock-infected cells. As for caspase-8 activity assay, a positive control was set via the TNF-α treatment. NA cells were treated with 100 ng/mL TNF-α for 6 h and then collected for caspase-8 assay.

### Real-time Quantitative PCR

To evaluate the transcription levels of target genes, NA cells infected with different RABVs were incubated for 2 days at 34°C. Cells were washed with ice-cold PBS and RNA was isolated using E.Z.N.A.^TM^ Total RNA Kit II and the RNase-Free DNase I Set (both purchased from Omega, United States). Reverse transcription was conducted using the Transcriptor First Strand cDNA Synthesis Kit (Roche, Germany) following the manufacturer’s protocols. Real-time quantitative PCRs (RT-qPCRs) were carried out in a CFX384 Real-Time System (Bio-Rad, CA, United States) using AceQ qPCR SYBR Green Master Mix (Vazyme) according to the manufacturer’s instructions. All experiments were run in triplicate.

A plasmid expressing the RABV genome was used as a standard to facilitate the quantification of viral messenger RNA (mRNA) and viral genomic RNA (gRNA) contained in infected NA cells (Zhao et al., 2010). Measured copy numbers of mRNA and gRNA were normalized to 1 μg of total RNA used for the experiment. The transcription levels of apoptosis-related genes (*Bax*, *Bcl-2*, *Caspase-3*, and *Caspase-9*) were determined and presented as fold-changes over those detected in sham-infected controls. The expression of the housekeeping gene glyceraldehyde-3-phosphate dehydrogenase was used as a reference in all experimental groups. Analyses of relative gene expression were conducted applying the 2^-ΔΔ*C*_T_^ method (Livak and Schmittgen, 2001). Primer sets were designed using Primer3 Input (Koressaar and Remm, 2007; Untergasser et al., 2012) and are listed in **Table 3**.

**Table 3:** Sequence of primers used to amplify the target and reference genes.

Amplicon	Origin	Forward primer (5′–3′)	Reverse primer (5′–3′)
Viral gRNA	Rabies virus	AGAAGAAGCAGACATCGTCAGTTG	GGAGACCACCTGATTATTGACTTTGA
N mRNA	HEP-Flury	TTTAGTCGGTCTTCTCCTGAGTCT	AATCTGCTCTATTCTATCCGCAATGT
	GD-SH-01	TACTCATCAAATGCGGTTGGTCAC	GCACATGCGGCAATAACTGTTG
P mRNA	HEP-Flury	GAGTCCAAATAGTCAGACAAATGAGGT	AGGAAAGTTGACCGAGACATAGGA
	GD-SH-01	CAGGTGTGACTCGTTTAGCTCATG	TGTCTGGCTCAACTAATAGCTGGA
M mRNA	HEP-Flury	AGAGGACAAAGACTCTTCTCTGCT	TGGAGTTAAGCCCGTATGTTCTCT
	GD-SH-01	TCCAAGGTAGGGTATGGTGTATCAA	AAGAGTCCTTGTCCTCCTCTGAC
G mRNA	HEP-Flury	GCCTTGATTGCCCTGATGTTGATAA	CATTTCTCCCTGTCCCTCCAAGAT
	GD-SH-01	ACGATAAATCCCTTCATTCGAGAGT	ATGGTGTAGTCATGGTTAGTAGAGC
L mRNA	HEP-Flury	TGTTGATGTCTGATTTCGCATTGTCT	AAGGGAACGCTCTTGACAGATGT
	GD-SH-01	GAGTCTGTCGTCTCACTGGATCA	GATGCCTTACCACCTCTCCAGATA
GAPDH mRNA	Mouse	CGTCCCGTAGACAAAATGGT	TTGATGGCAACAATCTCCAC
Bax mRNA	Mouse	TTGCTACAGGGTTTCATCCAGGAT	CTGTCCAGTTCATCTCCAATTCGC
Bcl-2 mRNA	Mouse	AACTCCCGATTCATTGCAAGTTGT	TAAGGACGGCATGATCTTCTGTCA
Caspase-3 mRNA	Mouse	GTGTATTGTGTCCATGCTCACGAA	CTCCCTGACAGCTTTCTCATTTGG
Caspase-9 mRNA	Mouse	GCATCTTTACCACCATCTCTGCC	TCGCTGTCAATTGTATGCTCTGTG


### Western Blotting

To investigate the expression of proteins involved in apoptotic signaling, Western blotting (WB) was conducted as described previously (Luo et al., 2016). Briefly, NA cells were infected with RABV and were incubated for 2 days at 34°C. At 2 dpi, the cell culture medium was discarded and cells were washed three times with ice-cold PBS before being lysed on ice with RIPA buffer (Beyotime) containing 1 mM phenylmethanesulfonyl fluoride (Beyotime), for 30 min. When analyzing the expression of cytochrome c, cytosolic and mitochondrial proteins were isolated using a Cell Mitochondria Isolation Kit (C3601, Beyotime) according to the manufacturer’s instructions. Total proteins were harvested and equal amounts of protein samples were boiled for 5 min in 6× SDS-PAGE loading buffer (Beyotime). Proteins were then separated in 12% SDS-PAGE gels, transferred to polyvinylidene difluoride (PVDF) membranes and incubated at 4°C overnight with primary antibodies directed against bax, bcl-2, PARP, cytochrome c, caspase-3, and caspase-9, respectively. β-Actin and COX IV were used as the loading controls, while the former for cytosolic proteins and the latter for mitochondrial proteins. After incubating membranes for 2 h at 37°C with goat anti-mouse or goat anti-rabbit secondary antibodies labeled with horseradish peroxidase, this enzyme’s activity was detected using BeyoECL Plus A and B solutions (Beyotime), according to the manufacturer’s instructions.

## Statistical Analysis

Unless otherwise stated, all data are represented as means ± standard derivations obtained from experiments performed at least in triplicate. Data were analyzed using GraphPad Prism 6 (GraphPad Software, United States). The statistical significance of differences between groups was verified using one-way ANOVA followed by Bonferroni’s multiple comparison test. For all tests, the following notations are used to indicate significant differences: ^∗^*p* < 0.05; ^∗∗^*p* < 0.01; ^∗∗∗^*p* < 0.001; ^∗∗∗∗^*p* < 0.0001, and ns, not significant (*p* > 0.05).

## Results

### Wild-Type GD-SH-01 Induces More Apoptosis than HEP-Flury While rHEP-shP Only Induces More Apoptosis at the Late Stage of Infection

Neuroblastoma cells infected with different RABVs were harvested to evaluate the degree of virus-induced apoptosis. In addition to GD-SH-01 and HEP-Flury, five rRABVs (rHEP-shN, rHEP-shP, rHEP-shM, rHEP-shG, and rHEP-shL) were used in this experiment. As shown in **Figures 1B,C**, remarkable apoptosis was observed in all groups except that infected with rHEP-shL, and the percentage of early stage apoptotic cells as wells as the late stage apoptotic/necrotic cells was significantly higher at 2 dpi in cells infected with GD-SH-01 than in those exposed to HEP-Flury and negative controls. What’s more, compared to HEP-Flury, much higher apoptotic rates were also obtained by infection with rHEP-shP, rHEP-shM, and rHEP-shG, which was also confirmed by the TUNEL assay (**Figure 1D**). The poor apoptotic ability of rHEP-shL may not result from its slightly weaker replicative ability, since rHEP-shP, rHEP-shM, and rHEP-shG induced remarkable apoptosis despite of the weaker replicative ability at an MOI of 2 (Tian et al., 2017). As shown in **Supplementary Figure S1** which presented the percentage of apoptotic cell at 1 dpi, of all five rRABVs, only rHEP-shM induced significantly higher apoptosis than HEP-Flury as GD-SH-01 did. Taking **Supplementary Figure S1** and **Figure 1C** together, we can see that compared to HEP-Flury, rHEP-shP induced comparable apoptosis at the early stage of infection (1 dpi), but induced more apoptosis at 2 dpi, indicating that rHEP-shP induced cell apoptosis in a time-dependent manner.

To exclude the possibility that the pro-apoptotic phenotype of rHEP-shP is just “artifact” attributable to the gene swapping between highly divergent strains, another rRABV, named as rGDSH-P (**Figure 2A**), was generated via a reverse genetics approach described previously (Tian et al., 2017). Analysis of the results of FCM in **Figures 2B,C**, it was shown that GD-SH-01 or rHEP-shP induced significantly higher cell apoptosis than that of HEP-Flury, rGDSH-P, or MOCK control. Of note, all virus-infected NA cells underwent significantly higher apoptosis than MOCK-infected cells. The cell viability assay further confirmed the higher apoptotic ability of GD-SH-01 and rHEP-shP (**Figure 2D**).

### GD-SH-01- and rHEP-shP Induce Apoptosis via Caspase-9 and Caspase-3 Activation

**FIGURE 3:**
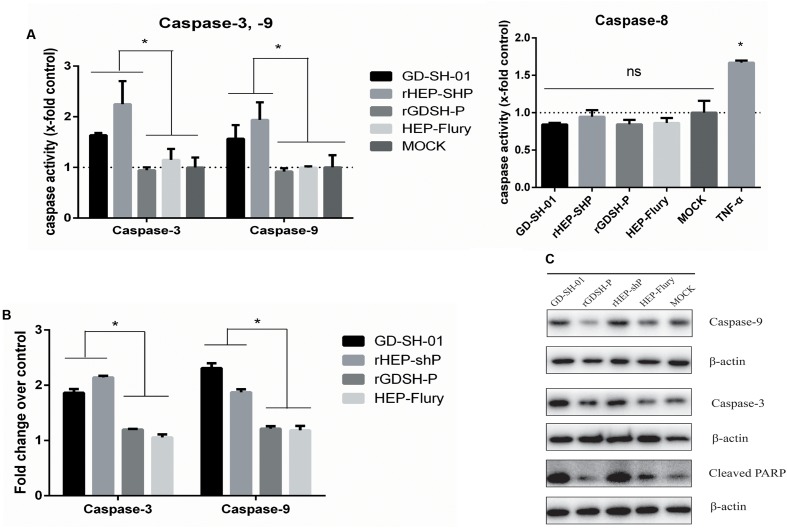
GD-SH-01 and rHEP-shP induce significantly greater apoptosis than HEP-Flury via the intrinsic apoptotic pathway. NA cells were infected with GD-SH-01, HEP-Flury, rHEP-shP, or rGDSH-P. **(A)** Caspase-3, caspase-8, and caspase-9 activities. **(B)** The relative expression of caspase-3 and caspase-9 as determined by means of RT-qPCR. **(C)** Protein contents of caspase-3, caspase-9, and cleaved PARP as measured by WB. β-Actin was used as a loading control. Data are represented as means ± SD of three independent experiments. One-way ANOVA followed by Bonferroni’s multiple comparison test. ^∗^*p* < 0.05.

To elucidate the mechanism underlying host cell apoptosis after an infection with GD-SH-01, rHEP-shP, rGDSH-P, or HEP-Flury, the activities of caspase-8, -9, and -3 were measured in RABV-infected NA cells. Our results show that caspase-9 and caspase-3 were activated in GD-SH-01- and rHEP-shP-infected cells, but not in HEP-Flury- and rGDSH-P-infected cells. In contrast, caspase-8 activation was detected in neither of the experimental groups (**Figure 3A**). In order to confirm the results obtained by means of caspase activity assays, RT-qPCR was conducted to quantify the transcription of both caspase-9 and caspase-3 in RABV-infected cells and negative controls. The results of these experiments were consistent with those described before: The expression of both caspase-9 and caspase-3 in cells infected with GD-SH-01 or rHEP-shP significantly exceeded that measured in HEP-Flury- or rGDSH-P-infected cells at 2 dpi (**Figure 3B**). Moreover, WB was conducted to evaluate the cellular content of caspase-9, -3, and cleaved PARP at protein level. Similarly, protein concentrations of caspase-9 and -3 in GD-SH-01- and rHEP-shP-infected cells were much higher than those in HEP-Flury- or rGDSH-P-infected cells, so was the contents of cleaved PARP, which served as a measure for executioner caspase activity (**Figure 3C**). Thus, apoptotic rates induced by GD-SH-01 and the rHEP-shP were higher than those induced by HEP-Flury and rGDSH-P, and were associated with the activation of caspase-9 and caspase-3, but not caspase-8.

### GD-SH-01 As Well As rHEP-shP Activates the Intrinsic Apoptotic Pathway during the Late Stage of Infection

**FIGURE 4:**
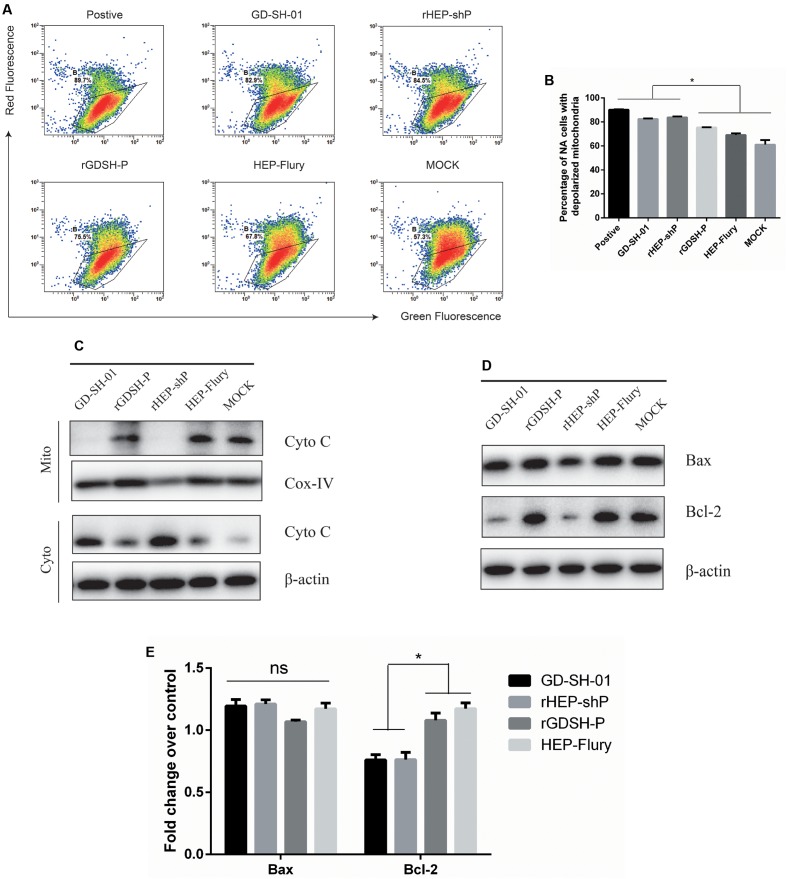
Analysis of activation of the intrinsic apoptotic pathway. NA cells were infected with GD-SH-01, HEP-Flury, rHEP-shP, or rGDSH-P. **(A)** Analysis of ΔΨm with FCM using JC-1 dye. A representative density plot is shown for each condition. Expression of ΔΨm expressed as the JC-1 monomer-to-polymer fluorescence ratio. **(B)** Analysis of the ΔΨm using chart plots. Protein contents of cytosolic and mitochondrial cytochrome c **(C)**, and bax and bcl-2 **(D)** were assessed by WB. COX-IV and β-actin were used as loading controls. **(D)** Relative expression of bax and bcl-2 as measured by means of RT-qPCR. Data are represented as means ± SD of three independent experiments. One-way ANOVA followed by Bonferroni’s multiple comparison test. ^∗^*p* < 0.05, ns, not significant.

Activation of caspase-9, but not caspase-8 in GD-SH-01- or rHEP-shP-infected NA cells at 2 dpi implied that these RABVs induced apoptosis via the intrinsic apoptotic pathway (**Figures 3A–C**). Then, we compared the ΔΨm, which is considered a marker of apoptosis (Zamzami et al., 1995), of NA cells infected with different RABVs by JC-1 staining. As shown in **Figures 4A,B**, compared to HEP-Flury-, rGDSH-P-, or MOCK-infected cells, significantly more severe mitochondria depolarization was seen in GD-SH-01- or rHEP-shP-infected cells, or CCCP-treated cells (positive control). This is consistent with our previous study which showed that a more severe loss/reduction of the ΔΨm occurs in GD-SH-01-infected cells when compared with HEP-Flury-infected cells at 2 dpi (Peng et al., 2016). Due to the key role of cytochrome c in the activation of the intrinsic apoptotic pathway, WB was conducted to assess the cytosolic and mitochondrial cytochrome c contents. As expected, compared with HEP-Flury- or rGDSH-P-infected cells or negative controls, cytosolic cytochrome c levels were remarkably higher in GD-SH-01- and rHEP-shP-infected cells, while mitochondrial cytochrome c levels were remarkably lower in GD-SH-01- and rHEP-shP-infected cells (**Figure 4C**). It indicated that along with the mitochondria depolarization, significantly more cytochrome c were released from mitochondria to cytosol in GD-SH-01- and rHEP-shP-infected cells.

### GD-SH-01 or rHEP-shP Infection Blocks Bax Activation and Downregulates Bcl-2 Expression

The above presented results demonstrate that GD-SH-01 or rHEP-shP activates the intrinsic apoptotic pathway during the late stage of infection. Since proteins pertaining to the bcl-2 family play key roles in the intrinsic apoptotic pathway (Cory and Adams, 2002), we wondered whether this also applies to the induction of apoptosis by GD-SH-01 or rHEP-shP. As shown in **Figure 4D**, concentrations of the pro-apoptotic protein bax were not notably affected by any RABV. However, the concentrations of anti-apoptotic protein bcl-2 were notably downregulated in cells infected with GD-SH-01- or rHEP-shP when compared with results obtained in HEP-Flury-, rGDSH-P-, or mock-infected cells. Those findings were consistent with those we got by means of RT-qPCR assays, as illustrated in **Figure 4E**.

### P Alone Does Not Suffice to Enhance Apoptosis

**FIGURE 5:**
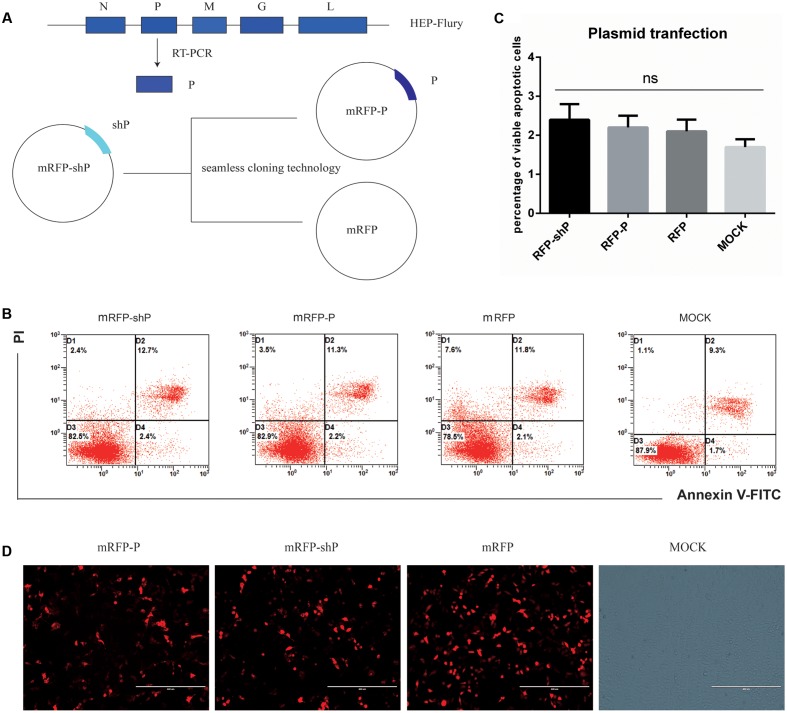
P of GD-SH-01 alone cannot induce detectable apoptosis. **(A)** Graphic illustration of constructed plasmids of mRFP-P, and control plasmid mRFP via seamless cloning technology on the background of mRFP-shP which was previously constructed. **(B)** NA cells were transfected with mRFP-shP, mRFP-P, and mRFP, respectively, and transfection without plasmid was used as a control. Apoptosis rates were determined 2 days post transfection by means of FCM. **(C)** The results in bar chart come from three scatter diagrams of each group. Data are represented as means ± SD of three independent experiments. One-way ANOVA followed by Bonferroni’s multiple comparison test. ^∗^*p* < 0.05, ns, not significant. **(D)** The efficacy of the transfection and expression levels of P was examined under a fluorescence microscope at 2 days post-transfection (representative images were presented here).

The fact that both GD-SH-01 and rHEP-shP significantly augment host cell apoptosis rates is indicative of the involvement of P in apoptosis induction. Therefore, we transfected cells with mRFP plasmids that carried P gene of GD-SH-01, HEP-Flury, or no P at all (**Figure 5A**). Apoptosis rates were determined at 2 dpi by means of FCM, and we found that there was no significant difference between any groups (**Figures 5B,C**). Before conducting the FCM, the target protein expression levels were assessed to ensure the proteins were indeed expressed and in a comparable level, as shown in **Figure 5D**, the red fluorescence intensities in mRFP-P- and mRFP-shP-transfected groups did not differ significantly, though a litter less than the control mRFP-transfected groups. Thus, despite results obtained after infections with GD-SH-01 or rHEP-shP, P alone cannot induce obvious apoptosis and seems to require one or more additional RABV elements to enhance apoptosis.

### The Enhancement of Host Cell Apoptosis by GD-SH-01 May Be Dependent on Viral RNA Synthesis But Not Viral Replication

**FIGURE 6:**
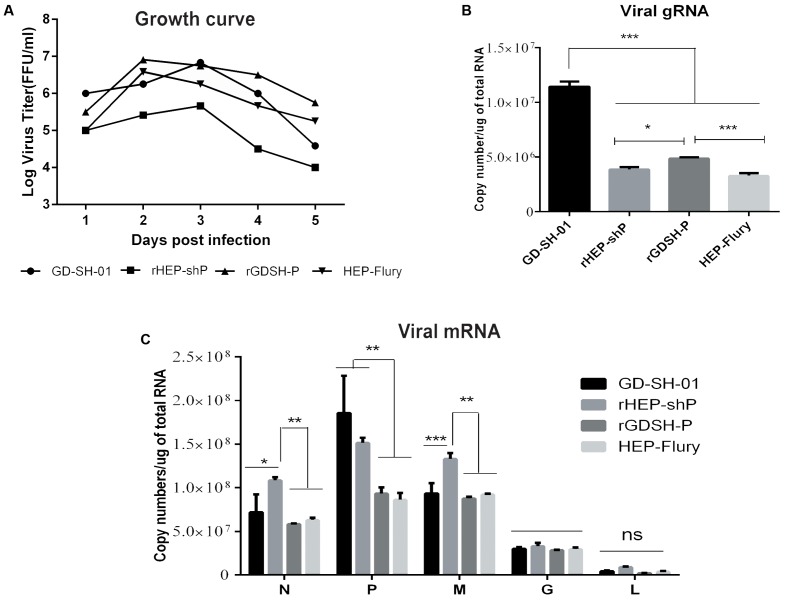
Viral RNA synthesis but not viral replication is required for the induction of host cell apoptosis by GD-SH-01 and rHEP-shP. NA cells were infected with GD-SH-01, HEP-Flury, rHEP-shP, or rGDSH-P. **(A)** Viral growth kinetics of the four RABVs. **(B)** Copy numbers of intracellular viral gRNA. **(C)** Copy numbers of intracellular viral mRNA. Data are represented as means ± SD of three independent experiments. One-way ANOVA followed by Bonferroni’s multiple comparison test. ^∗^*p* < 0.05, ^∗∗^*p* < 0.01, ^∗∗∗^*p* < 0.001, and ns, not significant.

The fact that the expression of P doesn’t suffice to augment apoptosis rates in host cells implies that the induction of apoptosis by viral pathogens depends on the presence of replicative viruses, as has been proposed previously (Thoulouze et al., 1997). We thus set out to verify whether viral replication was necessary for GD-SH-01 or rHEP-shP to trigger host cell apoptosis based on a recent study in which we found that both viral replication and G gene position were related in HEP-Flury-induced cell apoptosis (Mei et al., 2017). In this context, we determined viral growth kinetics and intracellular viral gRNA contents. As shown in **Figure 6A**, virus titers of GD-SH-01, rGDSH-P, and HEP-Flury exceeded those of rHEP-shP at 2 dpi by at least one order of magnitude, but rHEP-shP did induce apoptosis rates comparable to those caused by an infection with GD-SH-01 which were significantly higher than that measured in HEP-Flury- or rGDSH-P-infected cells. A similar result was obtained that the contents of viral gRNA in rHEP-shP-infected cells are significantly lower, while those in GD-SH-01-infected cells are not significantly lower, than those in HEP-Flury- or rGDSH-P-infected cells (**Figure 6B**). Consequently, the increase of host cell apoptosis did not depend on viral replication in GD-SH-01-infected cells and much less in cells infected with rHEP-shP.

We also assessed whether viral mRNA synthesis was needed for the augmentation of apoptosis rates as observed in GD-SH-01- and rHEP-shP-infected cells. As shown in **Figure 6C**, the N and P mRNA contents were significantly higher in the respective groups than after an infection with HEP-Flury or rGDSH-P. Since the contents of mRNA were at least 10-fold greater than that of gRNA (**Figures 6B,C**), the total RNA synthesis (including viral gRNA and mRNA) was significantly higher in GD-SH-01- and rHEP-shP-infected cells than in those exposed to HEP-Flury and rGDSH-P. These findings indicate that the viral RNA synthesis may be crucial for the strong induction of host cell apoptosis by GD-SH-01 and rHEP-shP.

## Discussion

Previous research has demonstrated that attenuated RABVs may induce apoptosis, while wt RABV strains were apparently less able to do so (Thoulouze et al., 1997; Yan et al., 2001; Prehaud et al., 2003). Findings in disagreement with this hypothesis have been reported (Ubol and Kasisith, 2000; Ubol et al., 2005; Yin et al., 2014; Prabhavathy and Palanivel, 2015). Meanwhile, in our published study, we confirmed the ability of wt strain GD-SH-01 to induce significantly stronger apoptosis than the attenuated strain HEP-Flury (Peng et al., 2016). These inconsistencies may possibly be attributed to the fact that the induction of apoptosis is strain-specific (Peng et al., 2016). Additional studies are required to evaluate the behavior of additional wt RABV strains to verify this hypothesis.

In the present research, we aimed at unraveling the mechanisms underlying apoptosis induction by GD-SH-01 and HEP-Flury. In order to identify which viral gene(s) account for the pro-apoptotic phenotype displayed by wt GD-SH-01, we compared this strain’s behavior with that of HEP-Flury and five rRABVs. Those rRABVs were constructed by replacing single genes of HEP-Flury with the corresponding genes of GD-SH-01. Our results show that rHEP-shP, rHEP-shM, and rHEP-shG present a stronger pro-apoptotic ability than HEP-Flury, which is similar to that of GD-SH-01. Previous research has demonstrated that M and G of RABV are key determinants of apoptosis: M of CVS partially targets mitochondria and induces mitochondrial apoptosis through caspase-dependent and caspase-independent pathways at the late stages of infection (Zan et al., 2016), and apoptosis induced by ERA is concomitant with G expression (Thoulouze et al., 1997; Prehaud et al., 2003), and our findings agree with these reports.

Analyses of cells infected with the aforementioned RABVs revealed that both GD-SH-01 and rHEP-shP induced significantly higher apoptosis than HEP-Flury, which indicates P of GD-SH-01 to play a role in the induction of host cell apoptosis. In order to clarify the molecular mechanisms triggered by P, we infected NA cells with GD-SH-01, HEP-Flury, the chimeric rHEP-shP, and rGDSH-P. According to the results of caspase activity assays, the intrinsic apoptotic pathway was activated in GD-SH-01- and rHEP-shP-infected cells. This conclusion is based on the fact that caspase-9 and caspase-3 were activated while caspase-8 was not. These findings were verified by means of RT-qPCR and WB. In general, the activation of both caspase-9 and caspase-3 strongly indicates mitochondria dysfunction, which leads to the release of cytochrome c (Jiang and Wang, 2000). In fact, we have previously shown that an infection with GD-SH-01 led to mitochondria dysfunction and subsequent apoptosis (Peng et al., 2016), which is also confirmed in the present study. To investigate whether members of the bcl-2 protein family are involved in this process, the pro-apoptotic bax and anti-apoptotic bcl-2 protein contents were determined in infected cells. Bax levels remained unaffected, while bcl-2 was downregulated in GD-SH-01- or rHEP-shP-infected cells. Obviously, the latter resulted in the stronger apoptosis. It should be noted that these findings are not consistent with those of CVS-11 replication inducing bax-related, caspase-dependent apoptosis in NA cells (Ubol et al., 1998). This disagreement may be partly attributed to the differences between the fixed strain CVS-11 and wt strain GD-SH-01. Since apoptosis is a complex physiological process, there may be other factors or pathways involved in the actions of GD-SH-01, such as the caspase-independent pathway, and further investigation need to be carried out to this end.

Analysis of percentage of apoptotic cells after transfection with plasmids expressing P of either GD-SH-01 or HEP-Flury revealed that neither variant of P is able to induce measurable host cell apoptosis in the absence of other viral elements. Although viral replication were reported to induce apoptosis (Thoulouze et al., 1997; Ubol et al., 1998), we found that, compared with HEP-Flury or rGDSH-P, the induction of enhanced apoptosis by GD-SH-01 or rHEP-shP may be dependent on viral RNA synthesis but not viral replication. These results may indicate P of GD-SH-01 to contribute to host cell apoptosis through viral RNA accumulation. Indeed, it has been reported previously that viral RNA content correlated with the degree of host cell apoptosis (Vassilev and Donis, 2000; Liu et al., 2014). The other possibilities may also should be taken into account in the further study, such as the viral protein levels in RABV-infected NA cells, especially the expression of M or G, which could be affected by the substitution of P from either GD-SH-01 or HEP-Flury in chimeric RABVs. A recent study proved P from a fixed RABV to interact with mitochondrial complex I and to provoke mitochondrial dysfunction (Kammouni et al., 2015). In addition, mitochondrial dysfunction has been shown to stimulate pro-apoptotic Fas signaling cascades (Li et al., 1998). Moreover, another study demonstrated that M of the Mokola virus, a lyssavirus of low pathogenicity, interferes with mitochondrial function and induces apoptosis (Gholami et al., 2008). Considering those reports and our own findings, we deduce that P of lyssaviruses like RABV may contribute to the induction of apoptosis by mediating functional disturbances in mitochondria. However, whether this applies for other RABV strains, either wt or attenuated ones, needs to be clarified in further investigations.

Here, we are providing data that broaden our understanding of the apoptosis induced by wt RABV at the molecular and cellular levels. Our results demonstrate for the first time that the P gene of wt GD-SH-01 contributes to the enhanced apoptosis induced by GDSH-01 and this process to be related to the activation of the intrinsic apoptotic pathway *in vitro*.

## Author Contributions

XG, QT, and YFW conceived and designed the study; QT, YFW, QZ, JL, HJ, BZ, MM, FW, YTW, JP, and TL performed the research; QT, YFW, QZ, and JL analyzed data; QT and YFW wrote the manuscript; XG and YL helped to revise and discuss in the manuscript; and all authors read and approved the final manuscript.

## Conflict of Interest Statement

The authors declare that the research was conducted in the absence of any commercial or financial relationships that could be construed as a potential conflict of interest. The reviewer SY and handling Editor declared their shared affiliation.

## Supplementary Material

**FIGURE S1 |** Wild-type GD-SH-01 induces significantly greater apoptosis than attenuated HEP-Flury at 1 dpi. NA cells were infected with GD-SH-01, HEP-Flury, or chimeric viruses. Cells in early stage apoptosis are Annexin V^+^/PI^-^, while dead cells correspond to those marked Annexin V^+^, chart plot of host cell apoptosis at 1 dpi. Data are represented as means ± SD of three independent experiments. One-way ANOVA followed by Bonferroni’s multiple comparison test. Comparing with HEP-Flury, ^∗^*p* < 0.05, ^∗∗^*p* < 0.01, and ^∗∗∗^*p* < 0.001.
