# Supplementary figures and images for: Phosphoprotein Gene Contributes to the Enhanced Apoptosis Induced by Wild-Type Rabies Virus GD-SH-01 In Vitro
Source: Front Microbiol. 2017 Sep 5;8:1697. doi: 10.3389/fmicb.2017.01697 (PMC5591860; doi:10.3389/fmicb.2017.01697)

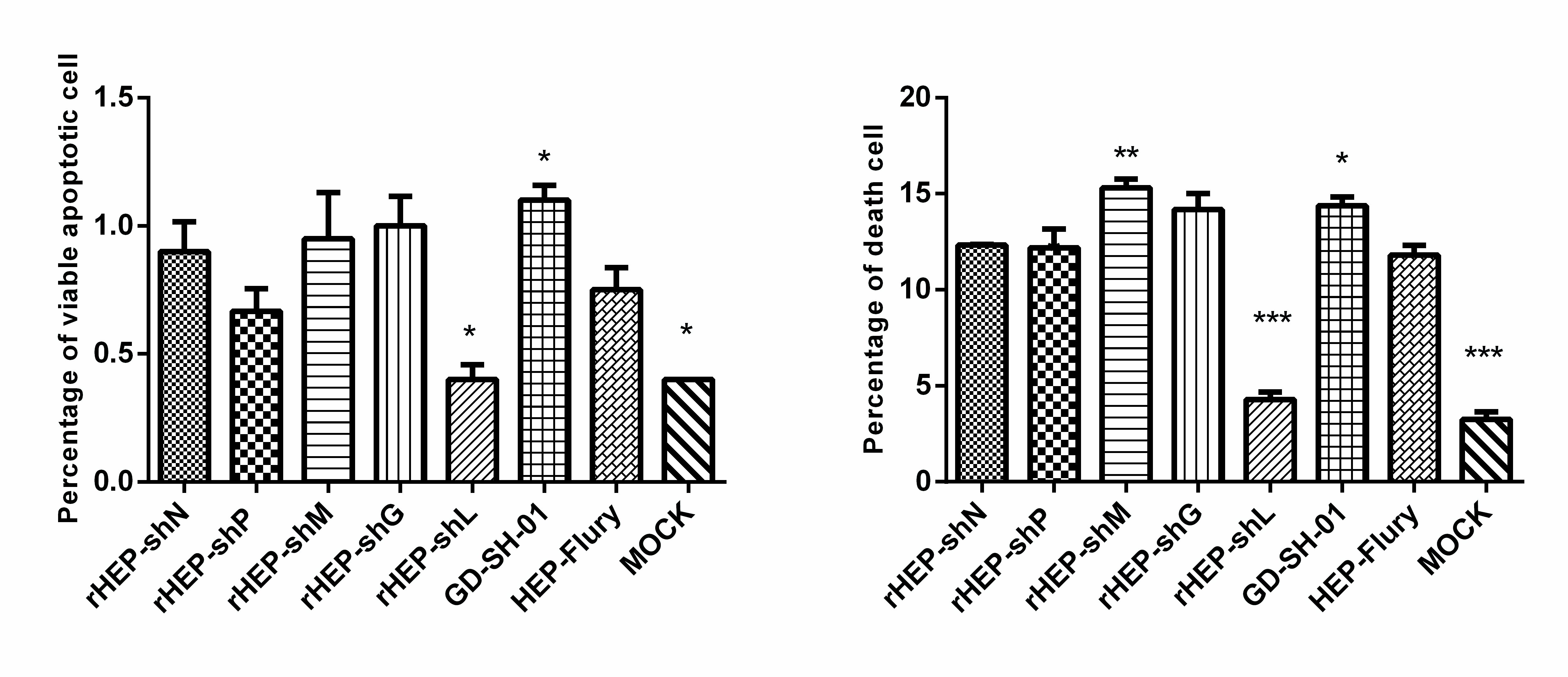

Supplement: Supplementary file 1 [file Image_1.TIF]
